# IL-31 Is Overexpressed in Lichen Planus but Its Level Does Not Correlate with Pruritus Severity

**DOI:** 10.1155/2015/854747

**Published:** 2015-02-03

**Authors:** Kalina Welz-Kubiak, Anna Kobuszewska, Adam Reich

**Affiliations:** Department of Dermatology, Venereology and Allergology, Wroclaw Medical University, Chalubinskiego 1, 50-368 Wroclaw, Poland

## Abstract

*Background*. Pruritus is one of the major features of lichen planus (LP); however, its pathogenesis remains largely unknown. *Objective*. The aim of our study was to analyze the role of IL-31 in the pathogenesis of pruritus in LP. *Materials and Methods*. The study group included 22 patients with LP. Control group consisted of 14 healthy volunteers. All subjects underwent thorough examination. Pruritus severity was evaluated with the visual analogue scale (VAS) and the 12-item Itch Questionnaire. IL-31 expression in the skin was assessed using semiquantitative immunofluorescence analysis. *Results*. Pruritus maximal intensity according to VAS was 6.5 ± 2.7 points and according to the 12-item Itch Questionnaire 6.9 ± 2.8 points. Lesional LP skin showed significantly higher IL-31 expression compared to healthy skin (*P* < 0.001). The most abundant immunofluorescence was observed within granular layer. However, there was no correlation between expression of IL-31 and pruritus intensity assessed according to VAS (VAS_max_: *ρ* = −0.08, *P* = 0.73), as well as 12-item Itch Questionnaire: *ρ* = −0.11, *P* = 0.65. *Conclusions*. Pruritus is a very common symptom of LP. For the first time we have demonstrated that IL-31 is overexpressed in the lesional skin of LP but its expression does not correlate with intensity of pruritus.

## 1. Introduction

Lichen planus (LP) is a chronic inflammatory disease involving both the skin and mucous membranes. This is relatively rare dermatosis, affecting about 0.5% of general population [1]. A wide variety of clinical manifestations and numerous subtypes of LP have been described, showing variable lesion configuration and morphology. However, LP is characterize by its typical histology with band-like lymphohistiocytic infiltrate at the dermoepidermal junction with vacuolar degeneration of the basal layer of the epidermis. Necrotic keratinocytes (civatte bodies or cytoid bodies) are extruded into the papillary dermis. Irregular acanthosis may result in a saw-toothed appearance of dermoepidermal junction. Hyperorthokeratosis may also be seen but is rather considered as a feature of lichenoid drug eruption [1].

The typical skin manifestation of LP involves the presence of shiny, polygonal, flat-topped, violaceous papules, and plaques. Reticulated white lines, termed “Wickham's striae,” are present on the papule top. It is believed that Wickham's striae result from focal hypertrophy of granular layer of the epidermis. Furthermore, LP lesions may appear as an isomorphic response to trauma (Koebner phenomenon). Skin changes most commonly arise on extremities, especially the flexural areas of wrists and ankles. Oral involvement is seen in about 30–70% of patients with LP. Lesions of oral LP most commonly appear as asymptomatic or tender, white, reticulated patches, or plaques (reticulated form) or as painful erosions and ulcers (erosive form). LP of the genitalia most commonly presents with pruritus or hyperalgesia and may lead to vaginal discharge or hemorrhage [1]. Importantly, itch is a cardinal subjective symptom of LP, which usually does not subside after common antipruritic treatment. Our previous studies indicated that pruritus is the most bothersome symptom of LP for the majority of patients suffering from this disease [2, 3]. However, pathogenesis and mediators of pruritus in LP are largely unknown.

Itch or pruritus is a cutaneous sensation different from pain. It is evoked by pruritogenic stimuli activating distinct subgroups of dedicated primary afferent C-fibers, including both histamine-sensitive and histamine-insensitive nonnociceptive polymodal nerve fibers, although nociceptive polymodal fibers may also be involved to some extent [4–6]. Keratinocytes, leukocytes, mast cells, fibroblasts, endothelial cells, and cutaneous nerves may produce several endogenous pruritogenic substances, including histamine, kinins, proteases, neurotrophins, some opioids, and cytokines [7]. Many of these mediators and modulators may directly activate the itch-sensitive C-fibers by specific receptors on nerve endings or can act indirectly by inducing the release of various pruritogenic mediators from other cells. Moreover, interactions among them can exacerbate and strengthen itch sensation to promote chronic pruritic skin diseases [8].

Despite the fact that the pathogenesis of LP is still not fully explained, it is known that LP results from a cell-mediated autoimmunity directed against keratinocytes of the basal layer, leading to the formation of subepidermal infiltrate initially composed of CD4+ lymphocytes, and, subsequently, also of CD8+ cytotoxic cells [1]. Activated lymphocytes produce many proinflammatory cytokines and it is very probable that some of them may be involved in induction of itch in LP. Some previous studies have shown that interleukin-31 (IL-31), a newly discovered, T-cell-derived, short-chain member of the alpha-helical family of IL-6 cytokines, and its receptor components: IL-31R*α* and OSMR, could be a key cytokine pathway responsible for itch accompanying some inflammatory skin conditions, such as atopic dermatitis [9–11]. The current study was undertaken to analyze whether IL-31 is also involved in the pathogenesis of pruritus in LP.

## 2. Material and Methods

### 2.1. Patients

The study group included 22 patients (10 males, 12 females, mean age: 50.1 ± 16.1 years, age range: 21–91 years) with LP treated at the Department of Dermatology, Venereology and Allergology of the Wroclaw Medical University between 2012 and 2014. A detailed characteristic of included subjects is demonstrated in Table 1. Control group consisted of 14 healthy controls (mean age: 61.5 ± 10.4 years, age range: 44–77 years) with no personal history of any allergy, atopic dermatitis (AD), LP, or other immune diseases.

### 2.2. Study Design

This study was approved by the Ethic Committee of the Wroclaw Medical University and was conducted in accordance with the principles of the Declaration of Helsinki. All participants signed an informed consent form prior to any study procedure. All patients underwent a careful dermatological examination. A specially designed questionnaire containing questions about demographic data, disease history, clinical features of itching, and administered therapy was completed by the dermatologists based on the detailed anamnesis and physical examination.

### 2.3. Assessment of Pruritus

The severity of pruritus was evaluated with the visual analogue scale (VAS) and the 12-item Itch Questionnaire. According to VAS patients were asked to estimate on the 10 cm long horizontal line the intensity of pruritus at the time of examination (VAS_current_), and at the time of maximal itching they had experienced within the last two weeks (VAS_max⁡_). The scores ranged from 0 (no itching) to 10 points (maximal itching) [12, 13]. The 12-item Itch Questionnaire is a validated instrument with questions about itching severity, frequency, and localization, as well as about disturbances in daily activities, sleeping, and psychological well-being caused by pruritus [14]. The scoring ranged from 0 (no itching) to 22 points (maximal itching).

Beside itch severity, we have also asked about various clinical features of itching such as diurnal/nocturnal variations of itching, most common factors aggravating and alleviating itching, itch quality, and descriptors.

### 2.4. Skin Biopsies

A 5 mm punch biopsies were taken from the lesional skin of 22 patients with histologically confirmed LP. Upon obtaining informed consent from healthy donors, punch biopsies were also taken from normal skin of 14 healthy volunteers. Cryosections of approximately 6 *μ*m were prepared from all skin tissue blocks and stored at the temperature of −70°C prior to staining. One fresh-frozen section from each patient was stained with hematoxylin and eosin (HE).

### 2.5. Immunofluorescence Analysis

Only samples with typical LP histology were included in the study. After unfreezing, cryosections were incubated with 4% formaldehyde in phosphate-buffered saline (PBS, pH 7.4) for 15 min at 4°C. After double washing with PBS (pH 7.4) they were warmed in microwave in 1 M citrate buffer (pH = 6.0) for 10 min, permeabilised on a shaker in 1% solution of Triton X-100 in PBS, blocked with 2% BSA, and incubated with antibodies against human IL-31 (anti-human biotinylated IL-31 antibody, dilution 1 : 50; Mabtech) overnight at 4°C. Next, slides were thoroughly washed in PBS and incubated with FITC Streptavidin (dilution 1 : 200; BioLegend) for one hour in dark chamber. After another washing, stained slides were mounted with mounting medium (Ultra Cruz Mounting Medium, Santa Cruz Biotechnology) and examined using Zeiss Axio Imager.A2 microscope (Zeiss, Germany). For negative controls, the primary anti-IL-31 antibody was replaced by 0.9% NaCl. The intensity of fluorescence was assessed according to following scale: 0: no reaction, 1: doubtful, 2: week positive, 3: moderately positive, and 4: strongly positive immunoreactivity. All experiments were repeated three times and the mean value for fluorescence intensity was used for further analysis.

### 2.6. Statistical Analysis

All data were analyzed statistically using* Statistica 10.0 *(Statsoft, Krakow, Poland). The *χ* ^2^ test with Yates correction, Student's *t*-test, Mann-Whitney *U* test, and Spearman rang correlation test were used where appropriate. *P* values less than 0.05 were considered statistically significant.

## 3. Results

### 3.1. IL-31 Expression

**Figure 1:**
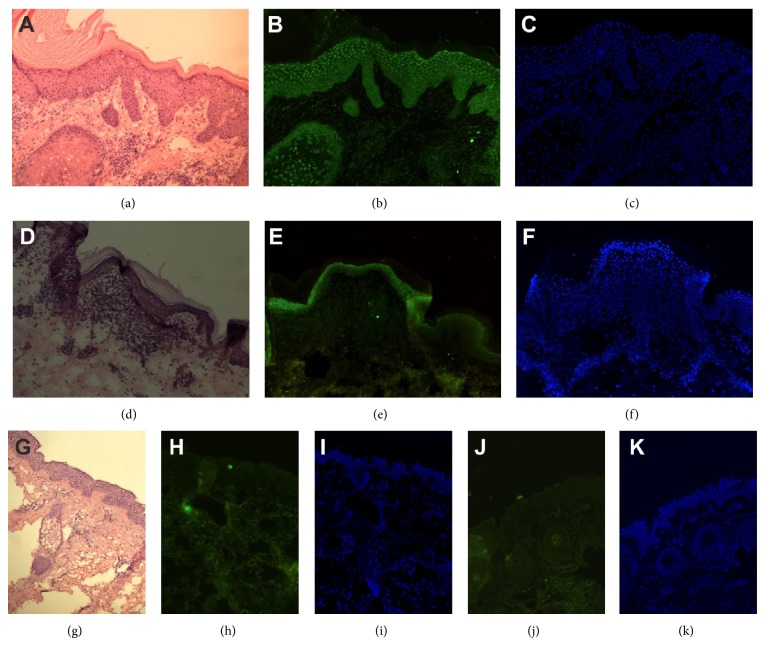
Expression of IL-31 was found in the entire epidermis of lichen planus lesions ((a)–(c): patient 1, (d)–(f): patient 2) in comparison to no expression in healthy skin ((g)–(i): volunteer 1, (j)-(k): volunteer 2) ((a), (d), and (g): hematoxylin/eosin staining of fresh-frozen section, (b), (e), (h), and (j): anti-IL-31 immunostaining, and (c), (f), (i), and (k): DAPI staining, original magnification ×100).

**Figure 2:**
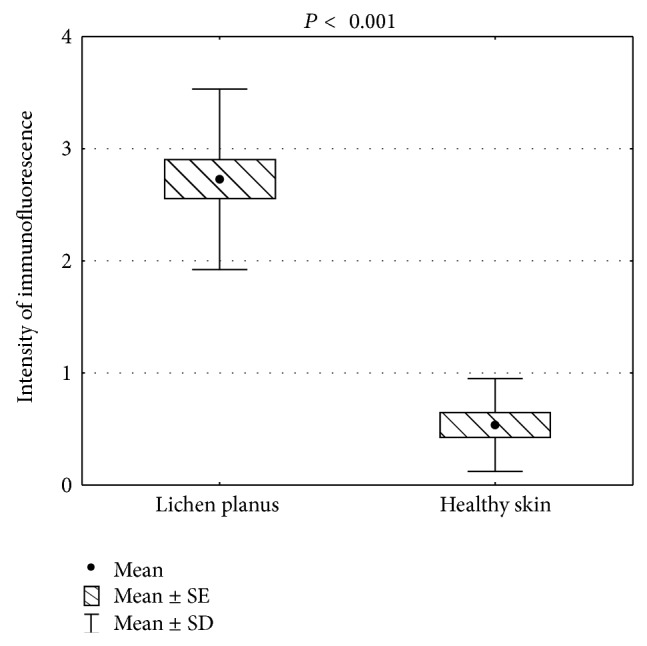
Comparison of the mean immunofluorescence intensity of anti-IL-31 staining between lichen planus and healthy skin (SE: standard error, SD: standard deviation).

Typical presentation of the expression of IL-31 in LP and healthy skin is demonstrated on Figure 1. All LP samples showed positive IL-31 immunoreactivity within all alive layers of epidermis. However, 8 (36.4%) patients demonstrated a strong homogenous IL-31 expression in all layers of epidermis (Figure 1(b)), while in 8 (36.4%) LP subjects the most abundant IL-31 immunoreactivity was observed within granular and spinous layers (Figure 1(e)), in 3 (13.6%) within granular layer, and in 3 (13.6%) within basal layer of the epidermis. The immunofluorescence within the inflammatory infiltrate in the dermis was very weak, if any (Figure 1). Lesional skin in LP showed significantly higher mean intensity of IL-31 immunofluorescence compared to healthy skin (2.72 ± 0.8 versus 0.54 ± 0.51, *P* < 0.001; Figure 2).

### 3.2. Pruritus Characteristics

Pruritus was observed in 21 out of 22 (95.4%) patients with LP. According to VAS the maximal intensity of pruritus ranged from 2 to 10 points (mean VAS_max⁡_: 6.5 ± 2.7 points), while the intensity during the examination ranged from 0 to 6 points (mean VAS_current_: 2.2 ± 1.8 points). Mean severity of pruritus according to 12-item Itch Questionnaire was 6.9 ± 2.8 points (range: 3–14 points). Pruritus severity was significantly correlated with the disease duration (VAS_max⁡_: *ρ* = 0.54, *P* = 0.02, 12-item Itch Questionnaire: *ρ* = 0.47, *P* < 0.05).

In most patients (*n* = 12, 57.1%) pruritus occurred in the evening and single itch episodes lasted from 1 to 10 minutes. In 3 subjects (14.3%) itch episodes lasted less than 1 minute and in remaining participants were longer than 10 minutes (*n* = 6, 28.6%). Pruritus was located mostly on the lower (*n* = 14, 66.7%) and upper extremities (*n* = 11, 52.4%) as well as on the trunk (*n* = 8, 38.1%). Majority of LP patients described pruritus as related to burning sensations (*n* = 15, 71.4%), less commonly to feeling of tickling (*n* = 4), prickling (*n* = 2), and warming (*n* = 2). Most participants considered itching as annoying (*n* = 12, 57.1%) and burdensome (*n* = 10, 47.6%).

### 3.3. IL-31 and Pruritus

There was no correlation between expression of IL-31 and pruritus severity assessed according to VAS (VAS_current_: *ρ* = −0.23, *P* = 0.34, VAS_max⁡_: *ρ* = −0.08, *P* = 0.73), as well as 12-item Itch Questionnaire (*ρ* = −0.11, *P* = 0.65). We also did not find any relationship between expression of IL-31 and location, duration, and any other analyzed features of pruritus (data not shown). In addition, no significant differences were observed between location of the most abundant immunofluorescence within different layers of epidermis and various analyzed characteristics of pruritus (data not shown).

## 4. Discussion

The biological functions of IL-31 are currently not well understood. It acts via a heterodimeric receptor composed of IL-31R*α* and oncostatin M receptor *β* and expression of IL-31 correlates with the expression of IL-4 and IL-13 suggesting that IL-31 is involved in Th2-mediated skin inflammation [15–17]. Several studies documented the importance of IL-31 in pruritus observed in atopic dermatitis (AD). Takaoka et al. [10] found that, in an animal model of AD–NC/Nga mice, long-lasting scratching behavior was accompanied by the increase of IL-31 mRNA expression which showed a good correlation with scratching counts. Intradermal injection of IL-31 in NC/Nga mice caused a gradual increase in long-lasting scratching about 3 h after administration followed by a gradual decrease for over 24 h after administration [18]. Similarly, repeated administrations of IL-31 significantly increased long-lasting scratching behavior parallel to overexpression of IL-31R*α* and oncostatin M receptor *β* in dorsal root ganglia [19]. When the repeated administrations of IL-31 were discontinued, IL-31R*α* expression decreased and reached the baseline level 2 days after the last dose of IL-31 [19]. Expression of IL-31R*α* was also found in human dorsal root ganglia neurons, largely in neurons that coexpress transient receptor potential cation channel vanilloid subtype 1 (TRPV1) [17]. Importantly, intraperitoneal administration of 10 mg/kg anti-IL-31 antibody reduced scratching behavior in NC/Nga mice but did not have any impact on dermatitis, further underlying the importance of IL-31 in AD pruritus [11]. However, data from human studies are less homogenous. IL-31 was significantly overexpressed in pruritic atopic compared with nonpruritic psoriatic skin inflammation and activated leukocytes from patients with AD expressed significantly higher IL-31 levels compared with control subjects [9]. Siniewicz-Luzeńczyk et al. [20] also observed increased serum IL-31 level in AD children than in healthy controls, but there was no correlation between serum IL-31 level and the disease severity or itch intensity. Similar results have been presented by other authors [21, 22]. In a very recent study by Sokołowska-Wojdyło et al. [23], it was suggested that some specific haplotypes of the IL-31 may be linked to the severity of AD. In addition, Hawro et al. [24] documented that IL-31 did not induce immediate itch in patients with AD or healthy volunteers but inconsistently induced delayed and mild itch, with no major differences observed in patients with AD and healthy controls. The late onset of IL-31-induced itch suggested the notion that IL-31 exerts in humans its pruritic effect indirectly via keratinocytes and secondary mediators, rather than through its receptors on cutaneous nerves [24].

The role of IL-31 in other pruritic skin diseases is far less understood. Narbutt et al. [25] demonstrated increased serum level of IL-31 in psoriatic patients which decreased upon UVB irradiation. However, similarly to our findings in LP, these authors also did not observe any relationship between pruritus intensity and IL-31 level in patients with psoriasis. In addition, increased serum IL-31 levels were noted in primary cutaneous T-cell lymphomas (CTCL) and it was even suggested that pruritus in CTCL may be related to IL-31 [26–28]. However, newer study did not confirm such suggestions, indicating that IL-31 might be involved in the pathogenesis of CTCL but is completely irrelevant for accompanying pruritus [29]. In addition, Nobbe et al. [30] also did not observe any significant relationships between IL-31 and IL-31R*α* cutaneous expressions and pruritus in pruritic skin conditions other than AD. Our observations in LP are in line with the results of above-mentioned studies. We also observed increased expression of IL-31 in lesional skin of LP, but its level correlated neither with itch intensity nor with any other clinical feature of pruritus. Higher expression of IL-31 in lesional skin of LP compared to healthy controls may suggest that this cytokine is involved in the LP pathogenesis; albeit, due to limited number of studied patients, our results should be interpreted with caution. Nevertheless, the exact role of IL-31 and mechanism, how it could induce inflammation in the skin, remains to be elucidated in the future. Interestingly, a recent study by Ko et al. [31] suggests that IL-31 might be relevant in uremic pruritus (UP), an example of systemic pruritus of to date unknown pathogenesis. Whether it is really a true itch mediator of UP or whether it rather represents an epiphenomenon like in a number of above-mentioned dermatoses needs also to be verified in the future.

In conclusion, for the first time we have shown an increased expression of IL-31 in lesional skin of LP patients. However, our data suggest that IL-31 is not involved in the pathogenesis of pruritus which is a cardinal symptom of LP. Further studies are needed in the future to exactly explore the role of IL-31 in the pathogenesis of LP.

## Figures and Tables

**Table 1:** Demographic and clinical characteristics of enrolled patients with lichen planus.

Gender (*n*):	
women	12
men	10
Age (years):	
mean ± standard deviation	50.1 ± 16.1
range	21–91
Type of LP (*n*):	
disseminated	13
localized	9
Disease duration (years):	
mean ± standard deviation	4.5 ± 8.9
range	0.08–37
Duration of the current exacerbation (months):	
mean ± standard deviation	4.6 ± 4.2
range	1–18
